# Rethinking self-injury recovery: a commentary and conceptual reframing

**DOI:** 10.1192/bjb.2019.51

**Published:** 2020-04

**Authors:** Stephen P. Lewis, Penelope A. Hasking

**Affiliations:** 1 Department of Psychology, University of Guelph, Canada; 2 School of Psychology, Curtin University, Australia

**Keywords:** Non-suicidal self-injury, self-harm, recovery

## Abstract

A growing body of research has focused on understanding what may contribute to cessation of self-injury. Although these efforts are of value, cessation represents just one component of self-injury recovery. Exclusive or primary focus on cessation may foster unrealistic expectations for those with lived experience of non-suicidal self-injury (NSSI). Accordingly, this commentary discusses the importance of expanding the concept of NSSI recovery beyond cessation in both research and clinical domains. We conclude by presenting a person-centred and non-stigmatising conceptual reframing of recovery.

Non-suicidal self-injury (NSSI), deliberate damage to one's body tissue in the absence of conscious suicidal intent,^1^ is a behaviour that is most often used to regulate intense or unwanted emotions.^2,3^ In community samples, 18% of adolescents, 13% of young adults and 5% of adults (aged 25+) report a history of NSSI,^4^ with approximately one-third persisting with the behaviour for longer than 1 year.^5^ Associated with psychological distress, mental health difficulties (e.g. depression, anxiety, eating disorders),^2,3^ interpersonal difficulties^2,3^ and subsequent suicide risk,^6^ NSSI can also leave physical scars that can represent both resilience and a source of shame for people who have self-injured.^7^

Given its many adverse consequences, researchers have recently focused on identifying factors that facilitate cessation of NSSI. In particular, researchers have sought to delineate individuals who currently engage in NSSI and those who report having stopped, with the aim of identifying targets for intervention.^5,8–12^ Clearly, there is merit in these efforts. Yet, this approach may inadvertently emphasise NSSI cessation, without equal attention to the many concerns those with lived NSSI experience report in the broader context of recovery. In keeping with a recent trend in the NSSI literature to offer more person-centred views of NSSI,^13–15^ the present commentary discusses issues inherent in limiting focus to NSSI cessation and offers a person-centred, non-stigmatising framing of recovery.

## Recent trends in NSSI recovery research

Notwithstanding the value of examining NSSI recovery, focus is often circumscribed to desistance of the behaviour. Indeed, this is typically the primary outcome measure in research.^5,8–12^ From a theoretical standpoint, NSSI has been situated within the context of recovery-based models in which desistance of a behaviour is the primary aim.^16^ For example, NSSI has been viewed in the context of the transtheoretical stages of change model, which considers individuals as ‘recovered’ (from the target behaviour) following a 6-month period of abstinence from that behaviour.^16–18^

In contrast to these views are those of individuals with lived NSSI experience, who have expressed concern that salient NSSI experiences may be neglected if the primary focus is on the behaviour.^19^ Indeed, individuals discuss a range of concerns (e.g. scarring, disclosure, coping) that extend well beyond NSSI disengagement.^6,7,20–23^ Taken together, the totality of experiences expressed by individuals with lived experience of NSSI may not consistently be represented in the extant literature. Moreover, emphasis on cessation of behaviour may inadvertently place NSSI in disease-based conceptualisations of ‘illness’. In particular, there has been a recent and growing movement away from pathologising behaviour, including NSSI.^24^ Notwithstanding the psychological and medical consequences of NSSI (e.g. distress, scarring), using disease-based language to describe NSSI can conflate illness with a behaviour, and exacerbate the already strong stigma associated with NSSI.^14^ Specifically, the tendency to conceptualise NSSI within models of infectious disease, including borrowing language from this domain (e.g. contagion), can have an ‘othering’ effect in distancing people who self-injure from those who are not ‘diseased’.^14^ This is reflected in recent research eliciting views from individuals with lived NSSI experience.^19^ Consistent with an emerging trend in the field, researchers and clinicians may find it more helpful to adopt a strengths-based and person-centred (rather than deficit- or disease-based) conceptualisation of NSSI and recovery.

## Moving beyond cessation

Cessation of NSSI represents just one element of an ongoing and multifaceted recovery process. Although many would not disagree that recovery is broader in scope, researchers typically focus on cessation of the behaviour,^5,9–12^ with the implicit argument that this is the desired outcome of any intervention effort. However, it is not uncommon for people with lived NSSI experience to mention ongoing NSSI thoughts or urges,^7,20^ learning new ways to cope with difficult emotions,^8,20,23^ disclosure-related concerns^22^ and coming to terms with scarring^7,21^ – even long after ceasing to self-injure.

Attenuated focus on complete NSSI cessation may result in people perceiving their own recovery as all-or-nothing. As ongoing NSSI thoughts and urges are common, beliefs that people can be ‘cured’ or fully removed from NSSI are unrealistic. Likewise, equating recovery with a single outcome and viewing cessation as the sole indication of success are unhelpful. Ultimately, considering cessation as ‘successful recovery’ detracts from the multifarious paths people inevitably have. Left with the impression that recovery is a linear path to cessation, individuals are prone to become discouraged (even when progress is made) or may view their own efforts as futile.

A more realistic expectation would be that many individuals *will* continue to experience thoughts and even urges to self-injure in the future. However, over time, these occurrences will abate in magnitude and frequency. Moreover, as individuals begin to find and utilise alternative strategies in lieu of NSSI, the pairing of NSSI with painful emotions should correspondingly dissipate. By acknowledging and ultimately adopting more realistic and holistic expectations, individuals are apt to feel encouraged over the course of their NSSI journeys.

## Reframing recovery

Following the above, we would encourage researchers and clinicians to adopt a broad, multipronged conception of recovery to account for a range of variables, including but not limited to NSSI cessation. Hence, we propose that consideration be given to how:

- people respond to difficult emotions and thoughts of NSSI (including coping responses);
- individuals adapt to and live with having NSSI scars;
- the process is non-linear and may involve setbacks (e.g. instances of NSSI);
- recovery may be an enduring (at times life-long) process; and
- other factors (e.g. disclosure, future coping, mental health difficulties) are germane.

By virtue of expanding beyond NSSI cessation, more realistic expectations can be fostered. This not only acknowledges the multitude of experiences people may have but stands to foster more resilience.

In keeping with the above, when working with people with lived NSSI experience, it might be more helpful for researchers and clinicians to strive to adopt and reflect back the precise language these individuals use when referring to recovery. As the lexis of recovery is commonplace in NSSI discourses,^17,18,25^ the term ‘recovery’ is bound to be used. Yet, alternative referents (e.g. journey, overcoming self-injury) may also be employed. Some people may even be resistant to using the term ‘recovery’ as it may position NSSI within disease-based (as opposed to behavioural) frameworks. As noted above, such framings have been rendered stigmatising by those with lived experience.^19^ Others may view the term recovery as conceptually ill-suited, as recovery is defined as a return to a normal or healthy state.^26^ Indeed, individuals may not view their experience as a return but more an experience in which they view themselves in a new light (e.g. more resilient);^7,23^ additionally, some may view this definition as inferring that people who self-injure are somehow ‘abnormal’. Ultimately, by using individuals' own language researchers and clinicians can avoid unnecessarily ‘correcting’ those who are arguably experts in *their* experience; further, this approach coheres with recommendations for discussing NSSI in assessment and related contexts.^3,27^

Nevertheless, irrespective of the phrasings used, it would be helpful if conversations could underscore and foster realistic expectations extending beyond NSSI cessation. This may necessitate ascertaining what individuals mean by the particular term they use. Doing so can help determine whether an individual's primary focus is on desistance of NSSI. Although desistance may have value for some people (e.g. acknowledging progress by the time elapsed since they last self-injured), it would be important to ensure that individuals view their trajectory realistically, cognisant of the manifold complexion of recovery (e.g. persistent urges, set-backs).

## Summary

Recent trends in the NSSI literature have seen the emergence of research on NSSI recovery, with a particular focus on factors related to cessation of the behaviour. Hopefully, it is apparent from our commentary that a primary focus on cessation in the context of self-injury could detract from the myriad experiences people have and may inadvertently lead to a sense of failure, as thoughts and urges (among other features) are apt to continue long after a person no longer self-injures. Instead, we call on researchers and clinicians to focus on how people respond to intense or unwanted emotions and whether the chosen strategies are meeting the desired aims. In this way, focus centres on individuals and their experiences, with realistic expectations about their own progress, while allowing the requisite space to adopt alternative strategies that will best serve the functions needed.

## About the authors

**Stephen P. Lewis**, PhD, is an associate professor in the Department of Psychology, University of Guelph, Ontario, Canada. **Penelope A. Hasking**, PhD, is an associate professor in the School of Psychology, Curtin University, Australia.
